# Progression of microstructural putamen alterations in a case of symptomatic recurrent seizures using diffusion tensor imaging

**DOI:** 10.1016/j.seizure.2012.03.015

**Published:** 2012-07

**Authors:** Jan S. Gerdes, Simon S. Keller, Wolfram Schwindt, Stefan Evers, Siawoosh Mohammadi, Michael Deppe

**Affiliations:** a Department of Neurology, University of Münster, Germany; b Department of Clinical Radiology, University Hospital Münster, Germany; c Wellcome Trust Centre for Neuroimaging, UCL Institute of Neurology, University College London, United Kingdom

**Keywords:** Serial imaging, Basal ganglia, White matter, Fractional anisotropy, Partial epilepsy

## Abstract

Microstructural alterations of the putamen were recently reported in patients with partial and generalized epilepsy disorders. However, it is unknown whether these alterations pre-exist or are secondary to recurrent seizures. Here we investigated the progression of putamen fractional anisotropy (FA) alterations in a case of recurrent psychomotor seizures using longitudinal diffusion tensor imaging (DTI) shortly *before* (DTI-1) and after a psychomotor seizure (DTI-2). We obtained FA values of a hypothesis-guided putamen region-of-interest (ROI) and seven exploratory ROIs. FA values from both DTIs were compared with reference values from 19 controls. Relative to controls, the patient's putamen FA was increased at DTI-1 (13% left putamen, 7% right putamen), an effect that was exacerbated at DTI-2 (24% left putamen (*p* < 0.05), 20% right putamen). In the exploratory ROIs we found FA reductions in the corticospinal tract, temporal lobe, and occipital lobe (*p* < 0.05) relative to controls at DTI-1 and DTI-2. In contrast to the putamen, all exploratory ROIs showed no relevant FA change between DTI-1 and DTI-2. These results suggest that recurrent seizures may lead to progressive microstructural putamen alterations.

## 1 Introduction

It has been recently reported that patients with cryptogenic temporal lobe epilepsy (TLE) and juvenile myoclonic epilepsy (JME) have inter-related increased diffusion tensor imaging (DTI)-derived measures of putamen fractional anisotropy (FA) and macroscopic putamen atrophy relative to age-matched healthy subjects.^1,2^ This unusual finding has been observed in other neurological disorders, including Huntington's disease^3,4^ and Susac's syndrome,^5^ but without sufficient neurological explanation.^3,4^ Given that the putamen and more generally the basal ganglia have a crucial role for the mediation of seizure propagation,^6^ it is important to understand how the putamen is architecturally affected in epilepsy. In patients with habitual epilepsy, it is difficult to confidently deduce whether neuroanatomical abnormalities pre-exist or are the consequence of recurrent seizures without monitoring the progression of brain structure and integrity during the course of serial imaging with simultaneous consideration of clinical variables, such as number and type of seizures, medication, etc. There are very few, if any, longitudinal DTI studies of the effects of recurrent seizures in patients with epilepsy syndromes that are not treatable by surgery. In almost all studies^7^ focus has been given to pre-/post-operative chronic partial epilepsy cohorts.^8,9^ DTI offers the opportunity to investigate microstructural changes not observable using conventional structural MRI,^10,11^ and quantification of such alterations^12^ may provide more information on progressive brain damage in epileptic syndromes. In the present study, we used serial applications of DTI to investigate the possible progression of gray and white matter alterations in a patient with symptomatic partial epilepsy, with a particular focus to investigate the stability or progression of putamen microstructural alterations in response to recurrent seizures.

## 2 Materials and methods

### 2.1 Case patient and healthy control subjects

A 48-year-old woman was found unconscious at home with deviation of the eyes and bite of the tongue. A cranial CT revealed a 2.2 cm × 2.7 cm measuring left parietal hemorrhage. During 20 months after onset of symptomatic epilepsy she had four complex-partial seizures with secondary generalization and three complex-partial seizures. Most frequently the patient presented with global aphasia, disorientation and mild paresis of the right arm. EEGs indicated a left temporal focus, occasionally with secondary generalization (for more detailed information see Table 1). We also studied a group of 19 neurologically and psychiatrically healthy age-matched controls (median 47 years, min 42, max 51). All subjects gave written informed consent and the local ethics committee approved this study.

### 2.2 Quantitative diffusion tensor imaging

The patient was admitted for DTI at two time points using a 1.5 T MR system (Gyroscan Intera T15, Philips Medical Systems, Best, The Netherlands). The time between the initial insult and the first DTI scan (DTI-1), and second scan (DTI-2), was 21 months and two days, and 23 months and one day, respectively. Time between the two scans was eight weeks and four days. For DTI we used single shot echo planar imaging (EPI) with 20 diffusion directions [two b-factors, 0 and 1000 s/mm^2^, TR = 9.3 s/TE = 89 ms, acquisition matrix: 128 × 128, voxel size: 1.8 mm × 1.8 mm × 3.6 mm (reconstructed to 2.0 mm × 2.0 mm × 2.0 mm for image processing), two averages]. DTI image processing was performed by using the “Münster Neuroimaging Evaluation System (EVAL)”.^12,13^ All time consuming calculations, e.g. eddy currents correction and normalizations, were carried out on a 64-processor computer (Sun Microsystems, Inc., Palo Alto). Diffusion-weighted images were corrected for eddy currents using a recently developed technique.^13^ The employed EVAL-DTI processing pipeline incorporated (i) structure adaptive smoothing^14^ and (ii) a multi-contrast image registration toolbox for the optimum spatial pre-processing of DTI data prior to statistical analysis.^15,16^ Registered FA images corresponded to the MNI coordinate space. For hypothesis-guided analysis of FA images, we generated a putamen region-of-interest (ROI) and for further explorative analysis seven other ROIs, including the whole white matter, corticospinal tract, corpus callosum, occipital lobe, frontal lobe and temporal lobe. These ROIs were created automatically by the EVAL pipeline on the output images from the registration toolbox for all patients and controls, as previously performed.^1^ For each of these ROIs mean FA was calculated.

### 2.3 Statistical analysis

We used a two-sample *t*-test including correction for multiple comparisons to test for differences in mean FA between patient's DTIs and controls. The FA values of the 19 normal controls have been tested by the Kolmogorov–Smirnov test for normality (*p* > 0.20). In order to assess intra-individual reproducibility of putamen FA, we compared putamen FA in a healthy control across 10 separate DTI measurements. We found that over the 10 DTI measurements, the control subject's putamen values were a mean of 0.127 with a standard deviation (SD) of ±0.009 for the right putamen and of 0.125 with a SD of ±0.008 for the left putamen (coefficient of variation CV = SD/mean × 100% = 7%).

## 3 Results

### 3.1 DTI-1

DTI-1 was performed three days after a complex-partial seizure (Seizure No. 7, Table 1), which occurred approximately 18 months after the initial hemorrhage. At this time, patient mean FA was increased in the left (+13%) and right (+7%) putamen relative to the mean putamen FA of controls (Table 2). The mean FA values of all exploratory white matter ROIs were reduced relative to controls. The most prominent reductions of FA relative to controls were observed in the left temporal lobe (−12%; *p* < 0.05) and occipital lobes (−18%; *p* = 0.001). Furthermore, significant reduction of ROI FA was observed in the whole white matter (*p* < 0.05), and bilaterally in the corticospinal tracts (*p* = 0.05, Table 2). Mean FA of the frontal lobes, corpus callosum and the right temporal lobe was also reduced (>1 SD relative to controls).

### 3.2 DTI-2

**Fig. 1:**
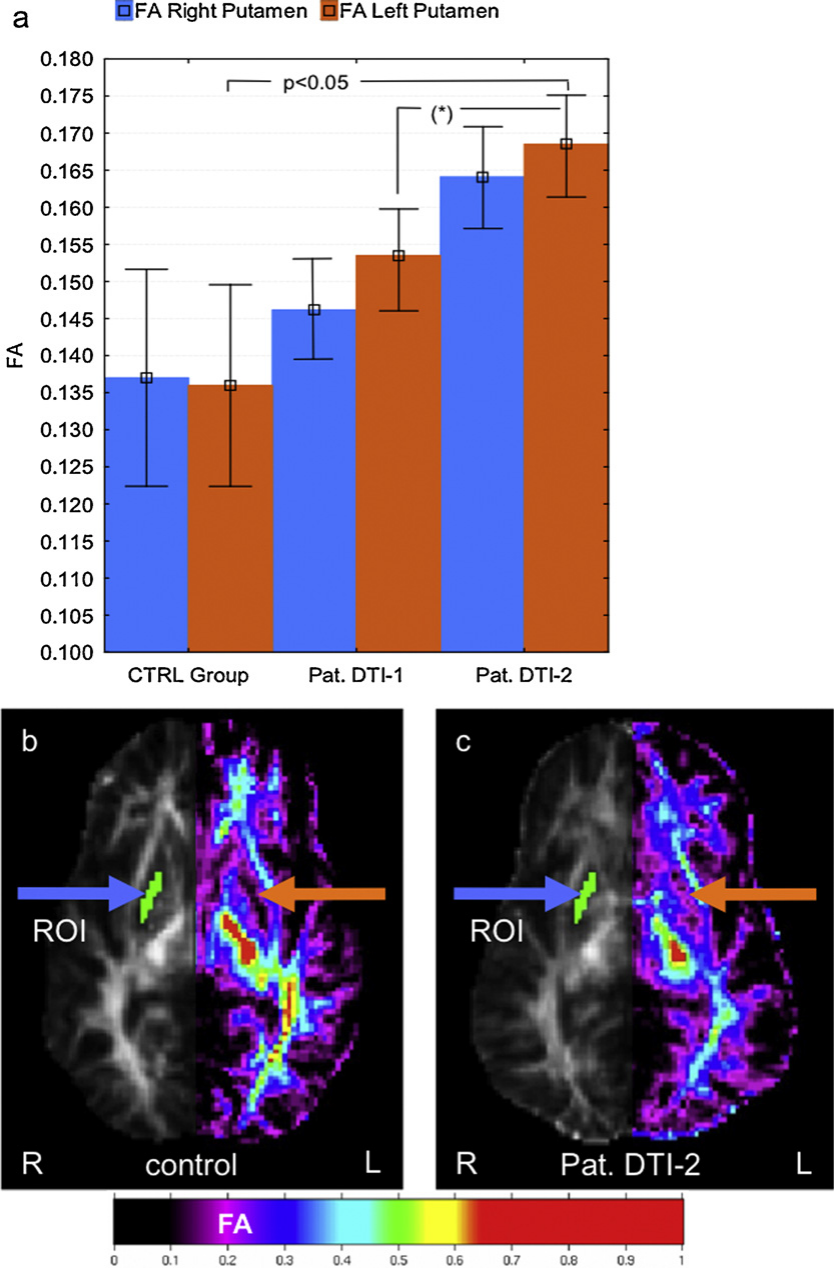
(a) Mean putamen FA of controls (CTRL Group) (whiskers represent the standard deviation) and patient's (Pat.) putamen FA of DTI-1 and DTI-2 (whiskers represent the estimated intra-individual reproducibility (7% CV) of FA). The increase of putamen FA between DTI-1 and DTI-2 was significant (*p* = 0.05) in the left putamen when compared with the mean left putamen FA of the healthy age-matched control group. (b) FA image of a healthy control. (c) FA image calculated from DTI-2 of the patient. The extent of the ROI is depicted for the right hemisphere; the color-coded FA is shown for the left hemisphere. The colored arrows indicate the position of the ROI for the left (orange) and the right (blue) hemisphere. *The increase of putamen FA values between DTI-1 and DTI-2 was beyond the estimated intra-individual reproducibility of the mean FA in the putamen ROI. (For interpretation of the references to color in the figure caption, the reader is referred to the web version of the article.)

Approximately six weeks after DTI-1 the patient's next seizure occurred (Seizure No. 8, Table 1). DTI-2 was acquired two weeks later. When comparing DTI-1 with DTI-2 the putamen FA was further increased (24% for the left putamen, ipsilateral to the lesion (*p* < 0.05) and 20% (*p* = 0.088) for the right putamen relative to controls). This increase of putamen FA between DTI-1 and DTI-2 was (i) beyond the estimated intra-individual variability (7% CV) and (ii) significant in the left putamen when compared with the mean left putamen FA of the controls. Progressive putamen FA increases in the patient are shown in Fig. 1. The exploratory white matter ROIs showed no relevant change of mean FA.

## 4 Discussion

The primary finding of this case is the progressive bilateral increase of putamen FA in a patient with a unilateral parieto-occipital hemorrhage, complex partial seizures and occasional secondary generalized seizures. The putamen is not causally linked with focal unilateral epilepsy disorders, but contributes to the modulation of seizure propagation after the principle epileptogenic focus has become active.^6^ In patients with TLE and JME, putamen macroscopic atrophy is directly related to pathologically increasing putamen FA.^1,2^ Furthermore, increasing putamen FA significantly correlates with the age of onset and duration of JME.^1^ In the present study, we observed a progressive putamen FA increase manifested as (i) an overall increase in the patient's putamen FA at DTI-1 relative to controls and (ii) a further putamen FA increase between DTI-1 and DTI-2 in the patient that exceeded the values observed in a randomly selected serially imaged healthy control.

As discussed in more detail previously,^1,2^ one explanation for the increase in putamen FA in patients with epilepsy may be due to increased levels of iron in patients with epilepsy that accumulate in the putamen, thus increasing the putamen FA and exacerbating a normal effect seen in healthy aging.^17^ Whether individual seizures increase such iron accumulation is unknown and warrants further investigation. An alternative explanation is that the atrophic gray matter within the putamen excessively constrains the space where numerous myelinated axons intersperse. Myelinated axons have a much greater FA compared to gray matter, and the spatial constriction of myelinated axons may lead to a localized increased anisotropy of water diffusion, given that this would occur beyond the voxel resolution of DTI.^1,2^ The putamen has been implicated in the normal inhibition of seizure generalization, and metabolic and structural alterations of the putamen have been reported in various epileptic syndromes.^6,18–20^ It is also important to note that increasing putamen FA is also observed in other neurological disorders including Huntington's disease^3,4^ and Susac's syndrome^5^ suggesting that this pathological alteration may not be specific to epileptic syndromes. The present results of increased putamen FA also extent findings by a cross-sectional study of increased FA in bilateral caudate nuclei and increased mean diffusivity values bilaterally in thalamus, putamen, and left caudate nucleus in patients with absence seizures.^21^

In summary, the findings of this case suggest that unilateral symptomatic partial seizures can lead to progressive microstructural changes of the putamen in both hemispheres. This is the first report that illustrates the dynamic of progressive microstructural subcortical alterations in a patient with a unilateral epilepsy disorder by comparison of DTI examinations performed shortly *before* and after a complex partial seizure. Future work should determine the aetiology of putamen FA increase in epileptic and other neurodegenerative syndromes.

## Conflict of interest statement

None of the authors has any conflict of interest to disclose. We confirm that we have read the Journal's position on issues involved in ethical publication and affirm that this report is consistent with those guidelines.

## Figures and Tables

**Table 1:** Seizure history with clinical details. LEV, levetiracetam; VPA, valproate; LTG, lamotrigin.

Seizure Nr.	Date	Clinical features	Seizure type	EEG	MRI or CT	Treatment
1	25.08.09	Deviation of the eyes, bite of the tongue	Symptomatic with secondary generalization	n.a.	Left parietal hemorrhage	LEV
						VPA
2	08.09.09	Aphasia, pronator drift right	Psychomotor complex-partial	Left temporal focus	Left parietal hemorrhage	LEV
						VPA
3	06.10.09	Aphasia, anisokoria deviation of the eyes to the left, incontinence for urine, no cyanosis	Complex-partial with secondary generalization, focal status epilepticus	Focal status epilepticus, bilateral frontal	Residual hemorrhage	LEV
						VPA
4	30.03.10	Arrest of speech	Complex-partial with secondary generalization	Left fronto-temporal focus with secondary generalization	Residual defect zone of the hemorrhage	LEV
5	22.06.10	Aphasia, apraxia, right-sided hemiparesis	Complex-partial with secondary generalization	Left temporal focus with secondary generalization	No recent alterations	LEV
6	11.01.11	Aphasia, slowing of the right arm	Complex-partial with secondary generalization	Focal status epilepticus, left temporal	No recent alterations	LEV
7	23.05.11	Disorientation, aphasia, pronator drift of the right arm	Psychomotor complex-partial	n.a.	No recent alterations	LEV
						LTG
8	09.07.11	Disorientation, aphasia	Psychomotor complex-partial	n.a.	Residual defect zone of the hemorrhage and HS	LEV
						LTG

**Table 2:** Columns represent mean FA and standard deviation (SD) of the controls and patient DTI-1 and DTI-2. Changes in % are calculated relative to the mean FA of controls. Significant (*p* < 0.05) changes are in bold. *p*-Values were corrected for multiple comparisons. ROIs are displayed in descending order according to changes in % of DTI-2.

ROI FA values									
	Controls		Pat. DTI-1			Pat. DTI-2			
	Mean	SD	Mean	Changes in % relative to controls	*p*	Mean	Changes in % relative to controls	*p*	Changes in % relative to DTI-1
Hypothesis-guided ROI									
FA left putamen	0.136	0.014	0.154	+13	0.225	0.169	**+24**	**0.031**	**+10**
FA right putamen	0.137	0.015	0.146	+7	0.550	0.164	**+20**	**0.088**	**+12**
Exploratory ROIs									
FA right temporal lobe	0.392	0.016	0.364	−7	0.104	0.371	−5	0.205	+2
FA frontal lobe	0.358	0.014	0.336	−6	0.157	0.337	−6	0.169	0
FA corticospinal tract	0.419	0.013	0.387	−8	0.031	0.390	−7	0.047	+1
FA all white matter	0.385	0.013	0.352	−9	0.024	0.357	−7	0.050	+1
FA corpus callosum	0.479	0.027	0.443	−8	0.204	0.442	−8	0.189	0
FA left temporal lobe	0.394	0.017	0.348	−12	0.015	0.345	−12	0.011	−1
FA occipital lobe	0.365	0.016	0.300	−18	0.001	0.311	−15	0.003	+4
